# Supplementary material for: Non-monotonic pressure dependence of high-field nematicity and magnetism in CeRhIn5
Source: Nat Commun. 2020 Jul 13;11:3482. doi: 10.1038/s41467-020-17274-6 (PMC7359027; doi:10.1038/s41467-020-17274-6)
Supplement: Supplementary file 1 — Supplementary Information [file 41467_2020_17274_MOESM1_ESM.pdf]

Supplementary Information
Non-monotonic pressure dependence of high-field nematicity and magnetism in $CeRhIn_5$

Toni Helm,[1,2,*] Audrey Grockowiak,[3] Fedor F. Balakirev,[4] John Singleton,[4] Jonathan B. Betts,[4] Kent R. Shirer,[1] Markus König,[1] Tobias Förster,[2] Eric D. Bauer,[5] Filip Ronning,[5] Stanley W. Tozer,[3] and Philip J. W. Moll[6,1]

[1] *Max Planck Institute for Chemical Physics of Solids, 01187 Dresden (Germany)*
[2] *Dresden High Magnetic Field Laboratory (HLD-EMFL), Helmholtz-Zentrum Dresden-Rossendorf, 01328 Dresden, (Germany)*
[3] *National High Magnetic Field Laboratory, Tallahassee, Florida 32310 FL (USA)*
[4] *National High Magnetic Field Laboratory Pulsed-Field Facility, MS-E536, Los Alamos National Laboratory, NM 87545 (USA)*
[5] *Los Alamos National Laboratory, Los Alamos, New Mexico 87545 NM (USA)*
[6] *Laboratory of Quantum Materials (QMAT), Institute of Materials (IMX), École Polytechnique Fédérale de Lausanne, 1015 Lausanne (Switzerland)*

(Dated: This manuscript was compiled on June 16, 2020)

Keywords: Heavy fermion superconductor; Nematicity; Quantum criticality; Diamond anvil pressure cell; High magnetic field; Focused ion beam microstructure

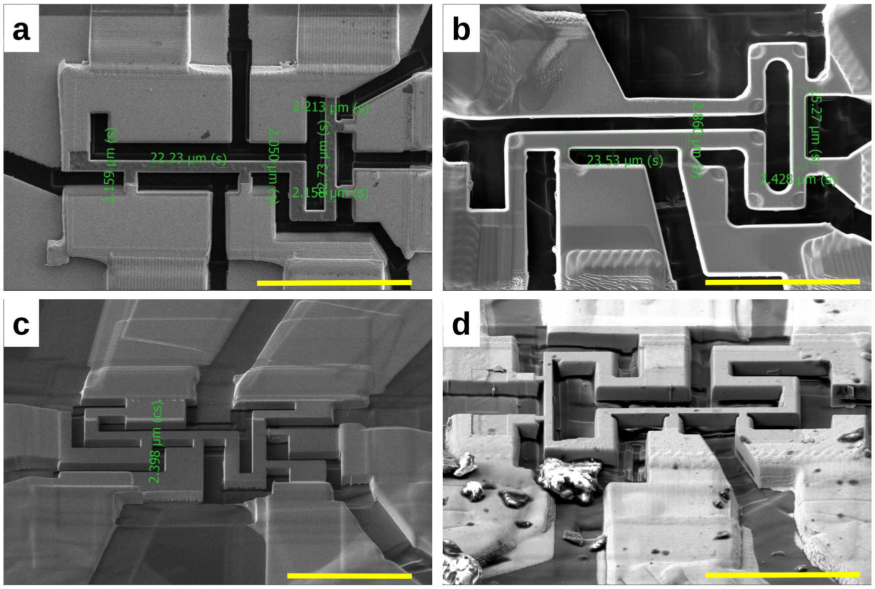

Supplementary Fig. 1. **Example microstructures.** L-shaped devices were fabricated as described in the methods section by the application of FIB. **a** Sample 1, **a** Sample 2, and **c** sample 3, all used in this study. In **d** we show a fourth device that had been pressurized to $p = 12\,\text{kbar}$. After depressurization and the removal of the pressure cell gasket it remained completely unharmed and fully functional (white pieces are debris of the plastic gasket). Yellow scale bars represent a distance of 30 μm.

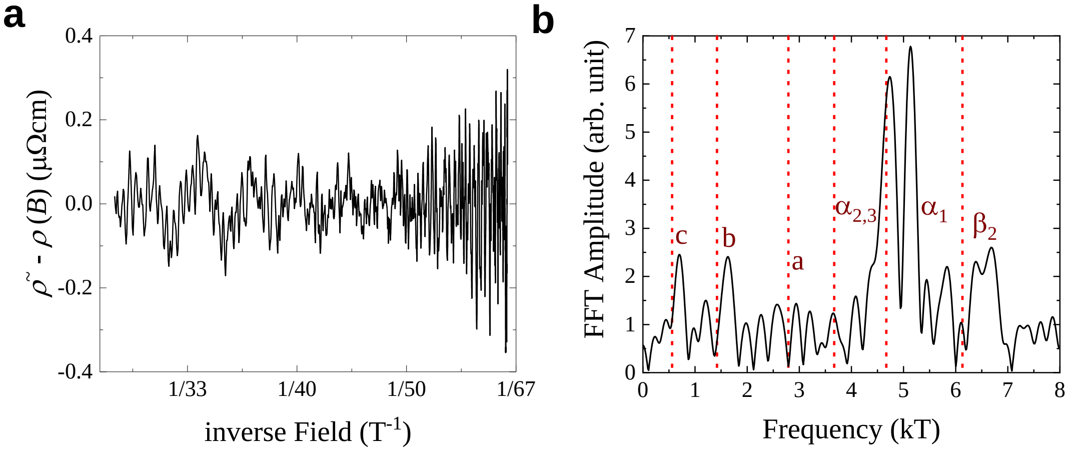

Supplementary Fig. 2. **Magnetic quantum oscillations.** Here we review the magnetic quantum oscillations (MQOs) observed in the resistivity of Sample 1, i.e. Shubnikov-de Haas oscillations at ambient pressure. **a** SdH oscillations from **Fig. 1e and f** of the main text obtained from Sample 1 at ambient pressure, $T = 0.5\,\text{K}$, and a tilt angle of $\theta = 20°$. Vertical red lines mark frequencies published from previous dHvA measurements at $\theta = 0°$ [1]. **b** shows the fast Fourier transform (FFT) spectrum of the oscillation data above 50 T after background subtraction. Multiple frequencies are evident. We compare the observed frequencies from the transport devices on diamond anvils with those measured in previous low-field de Haas-van Alphen (dHvA) experiments for $B||c$ [1]. The frequencies match well, and the small discrepancies are consistent with the differing alignment of the magnetic field for the transport measurements (20° off the c-axis). From previous dHvA oscillation studies [1] we know that the effective masses grow with increasing pressure, until they diverge at the QCP. Hence, it is no surprise that there are no oscillations discernable for higher pressures in our data.

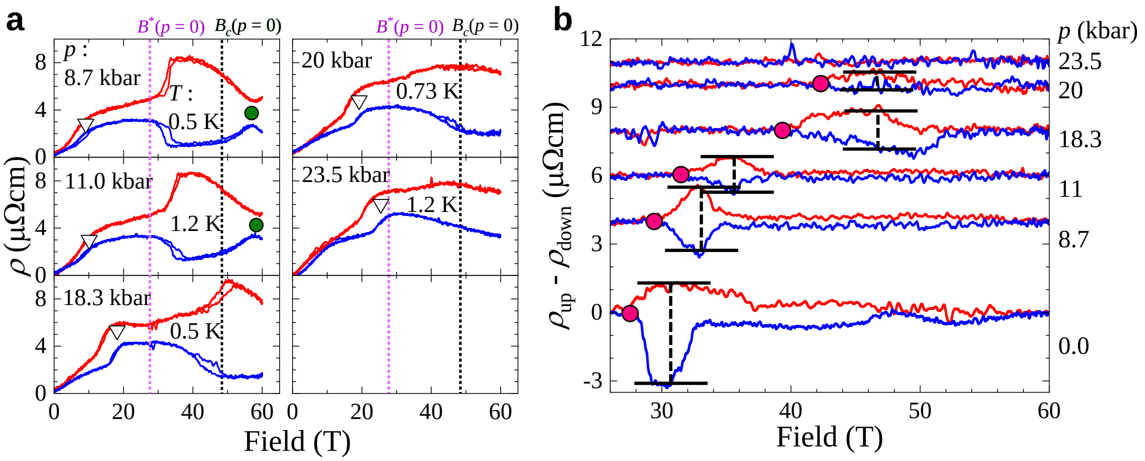

Supplementary Fig. 3. **Magnetoresistivity of sample 3.** To ensure reproducibility of the results, the entire series of experiments was carried out on three, nearly identical devices. All three samples were cut at the same angle from $CeRhIn_5$ crystals. Two samples were alternatingly measured inside the same setup attached to two different probes to avoid any systematic errors due to the vibrational and / or electrical properties of a single probe. The experiments have been performed in different magnets, and the quality and reproducibility of the data between magnets is evidenced by the almost seamless interleaving of relevant features in the three samples. The main results reported in the manuscript are well supported by all three samples. **a** Magnetoresistivity curves recorded for sample 3 (with a similar design as for sample 1 and 2) at five different pressures; Red and blue correspond to $I||a$- and $I||b^*$, respectively. The magenta and black dotted lines mark the nematic onset field $B^*(p=0)$ and the AFM suppression field $B_c(p=0)$ at ambient pressure, respectively. For 8.7 and 11 kbar B_c is highlighted by green circles. The hollow triangles mark the metamagnetic transition field B_M. **b** Difference between up- and down-field sweeps shown in **a**. Black bars highlight the maximum difference between curve couples, Δ associated with the hysteresis due to the nematic high-field phase. The nematic critical field B^* is marked by pink circles. Note: there are slight differences between the samples in the absolute values that are within the experimental errors. These are related to differences in the alignment of the devices of a few degrees.

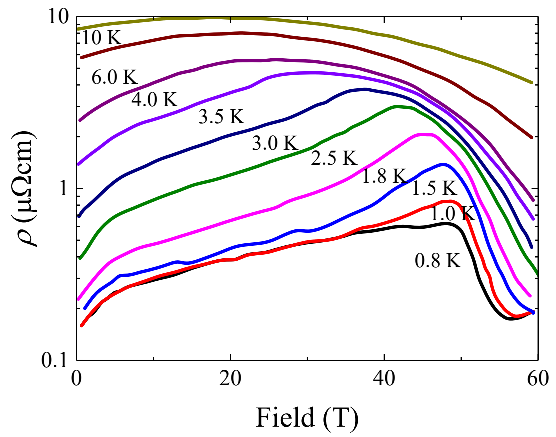

Supplementary Fig. 4. **Identifying the AFM transition field in the magnetoresistivity response.** The magnetic phase diagram, and the role of magnetic scattering in the anisotropic transport coefficients, is typically complicated in frustrated magnets and currently defies a quantitative analysis. Previously we have shown that for $CeRhIn_5$ the AFM suppression field B_c can be traced by magnetoresistivity. Here, we show the in-plane magnetoresistivity as a function of field $B||[010]$ for different temperatures (data from Ronning et al. [2]). The transition into the AFM state is clearly visible as a kink in the curve moving to higher fields at lower temperatures. At the lowest temperatures, the signature turns into a step-like transition.

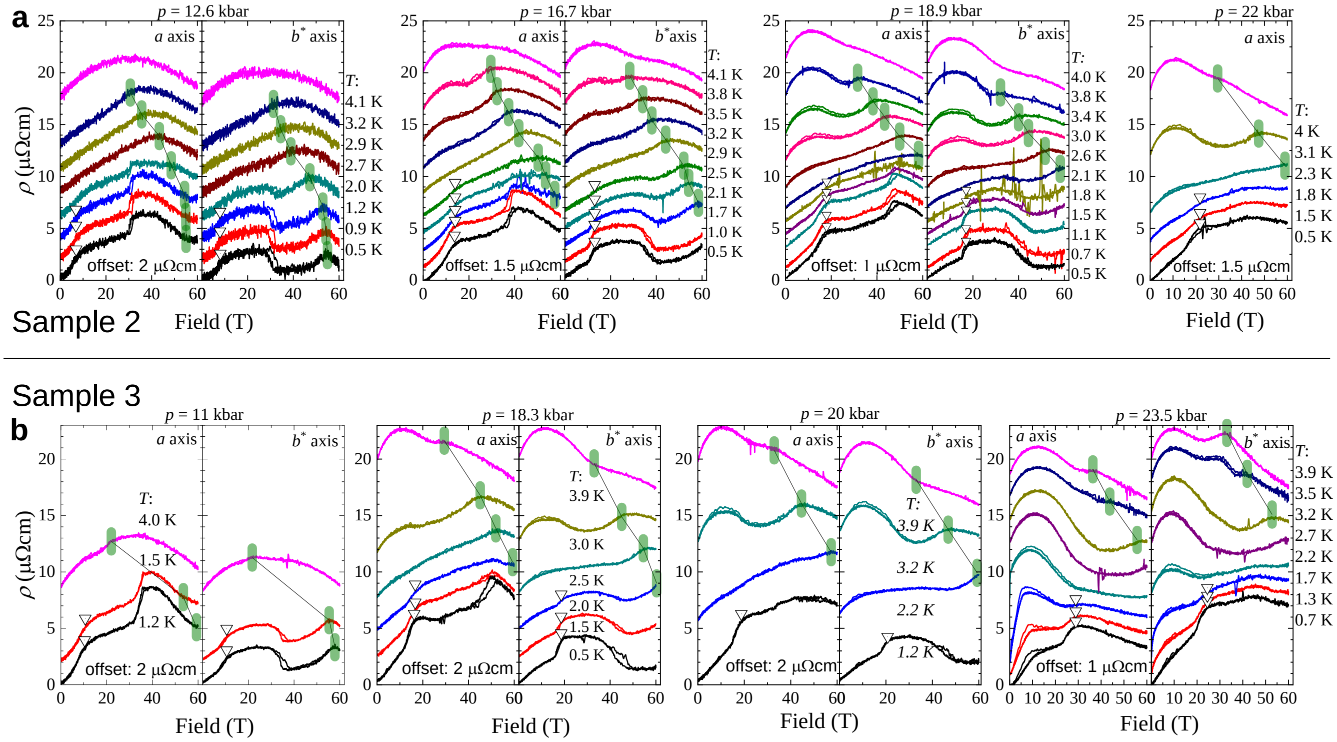

Supplementary Fig. 5. **Pressure-evolution of the AFM suppression field** B_c**. a and b** Magnetoresistivity recorded at various temperatures for sample 2 at pressures of 12.6, 16.7, 18.9, and 22 kbar, for sample 3 at $p = 11$, 18.3, 20, and 23.5 kbar. Curves are shifted by a constant offset for clarity. **Left and right panels**, respectively, exhibit curves for $I||a$ and $I||b^*$; the latter has a lower resistivity at high field due to contributions of c axis resistivity (see main text). Vertical green dashes mark the local feature with high curvature (i.e. a max/min in the 2nd derivative) we associate with the AFM suppression field B_c. Hollow triangles mark the metamagnetic transition at B_M emerging at low temperatures. While the increase of $B_c(p)$ in the low-pressure region is directly evident from the data, its continuation across p_c is difficult to confirm at the same confidence, as $B_c(p)$ at zero temperature leaves the experimentally accessible field window of 60 T. As discussed in the main manuscript, we rely on the extrapolation of the high-temperature values of $B_c(p, T)$. Owing to experimental variations, it was not possible to stabilize the exact same temperatures for different pressures. This allows to directly trace the evolution of the break-in-slope that is known from zero-pressure measurements to correspond to $B_c(p = 0, T)$. A gradual and continuous evolution across p_c is directly evident from the raw data. Even without any extrapolation it becomes self-evident that the magnetism crosses through $p = p_c$ unhindered at high magnetic fields.

Note: Sample 2 exhibited enhanced electronic noise during the initial low-pressure runs. We were able to improve on that for pressures above 12.6 kbar.

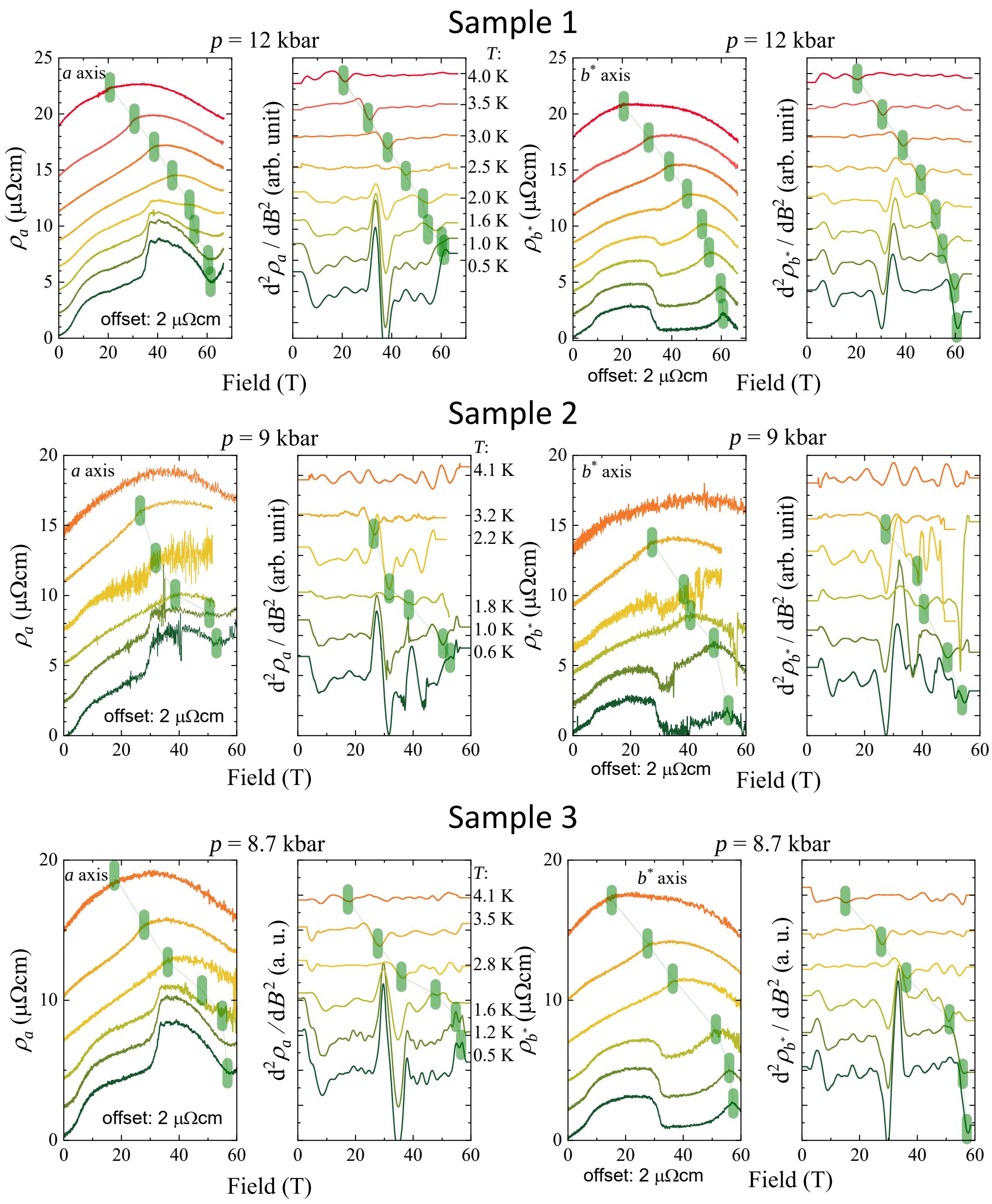

Supplementary Fig. 6. **Determination of** B_c **by second derivative.** Magnetoresistivity for Sample 1, 2 and 3 recorded for initial low pressures at different temperatures. Curves are shifted by a constant offset for clarity. **Left and right sides** are the field-down sweeps for $I||a$- and $I||b^*$-direction, respectively. **Right panels** show second derivatives of the data in the panel to their left. Green vertical dashes mark the AFM suppression field B_c. A feature with a maximum/minimum in the second derivative can be traced that shifts to lower field as temperature is decreased.

In order to self-consistently determine the resistive signatures we associate with the suppression and onset of magnetic order at high field and pressure, we rely on the second derivative of each magnetoresistivity curve. Here, we present only field down-sweep traces and their respective second derivatives, due to the lower noise level during the slower down-sweep of the pulse. Given the noise in pulsed fields, the second derivative was smoothened significantly. We removed noise from the raw signals using the wavelet transform package provided by OriginPro (More detailed information can be found for example in Refs. [3, 4]). The noisy signal is first decomposed using multi-level wavelet decomposition. We apply Daubechies or Haar wavelet algorithms and set a treshold for the coefficients related to the wavefunction, i.e. the detail coefficients. Finally, the approximation coefficients (related to the scaling function used) and altered detail coefficients are used to reconstruct the signal. Thereafter, we apply a Savitzky-Golay-filter smoothing before differentiation. Note: Sample 2 exhibited enhanced electronic noise during the initial low-pressure runs. We were able to improve on that for pressures above 12.6 kbar.

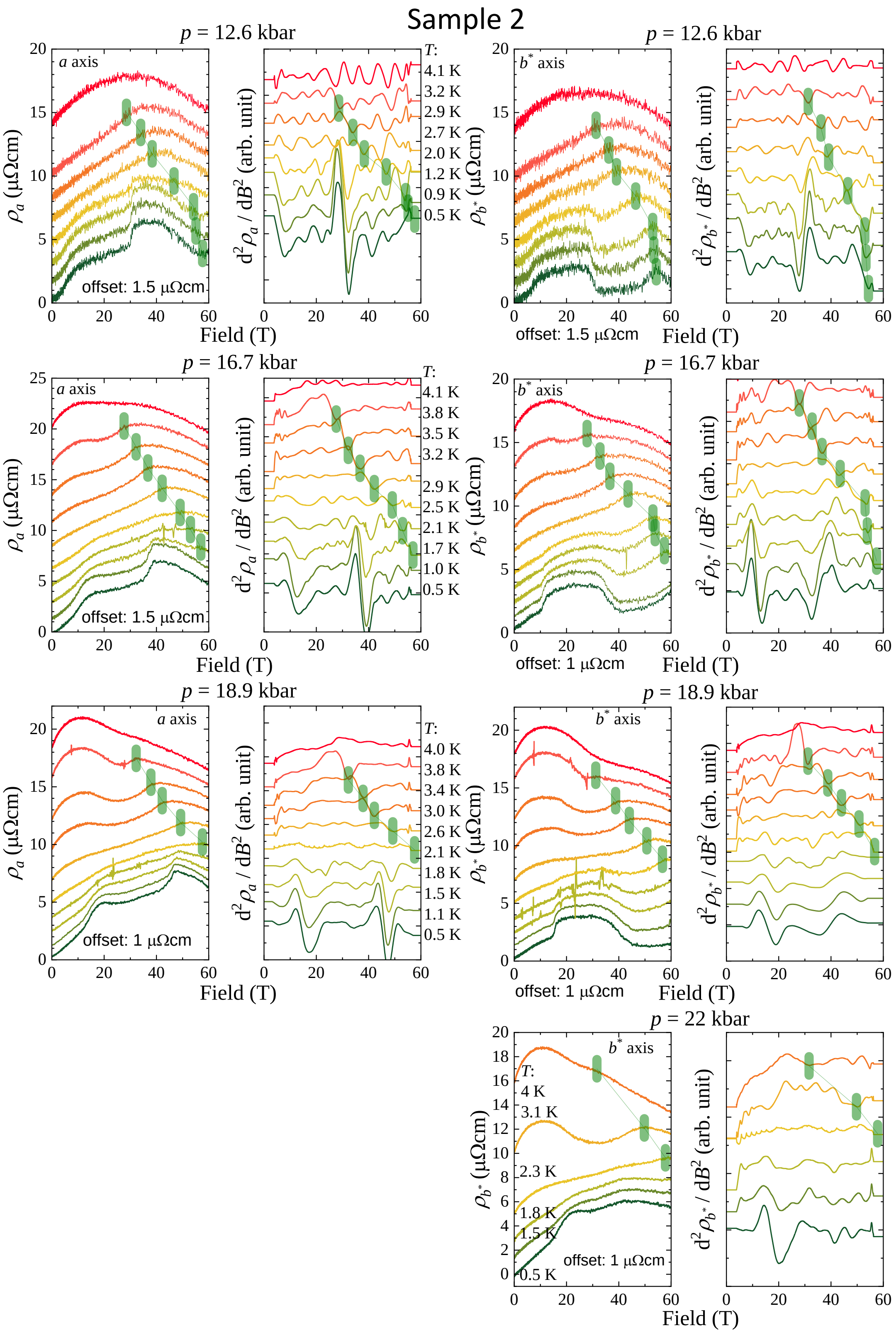

Supplementary Fig. 7. **Determination of** B_c **by second derivative.** Magnetoresistivity for Sample 2 recorded for four pressures at different temperatures. Curves are shifted by a constant offset for clarity. **Left and right sides** are the field-down sweeps for $I||a$- and $I||b^*$-direction, respectively. **Right panels** show second derivatives of the data in the panel to their left. Green vertical dashes mark the AFM suppression field B_c. Hollow triangles mark the sholder-like feature associated with the metamagnetic transition. Note: For 22 kbar we only recorded the b^* channel due to broken leads, those had been recovered afterwards for higher pressures. Sample 2 exhibited enhanced electronic noise during the initial low-pressure runs. We were able to improve on that for pressures above 12.6 kbar.

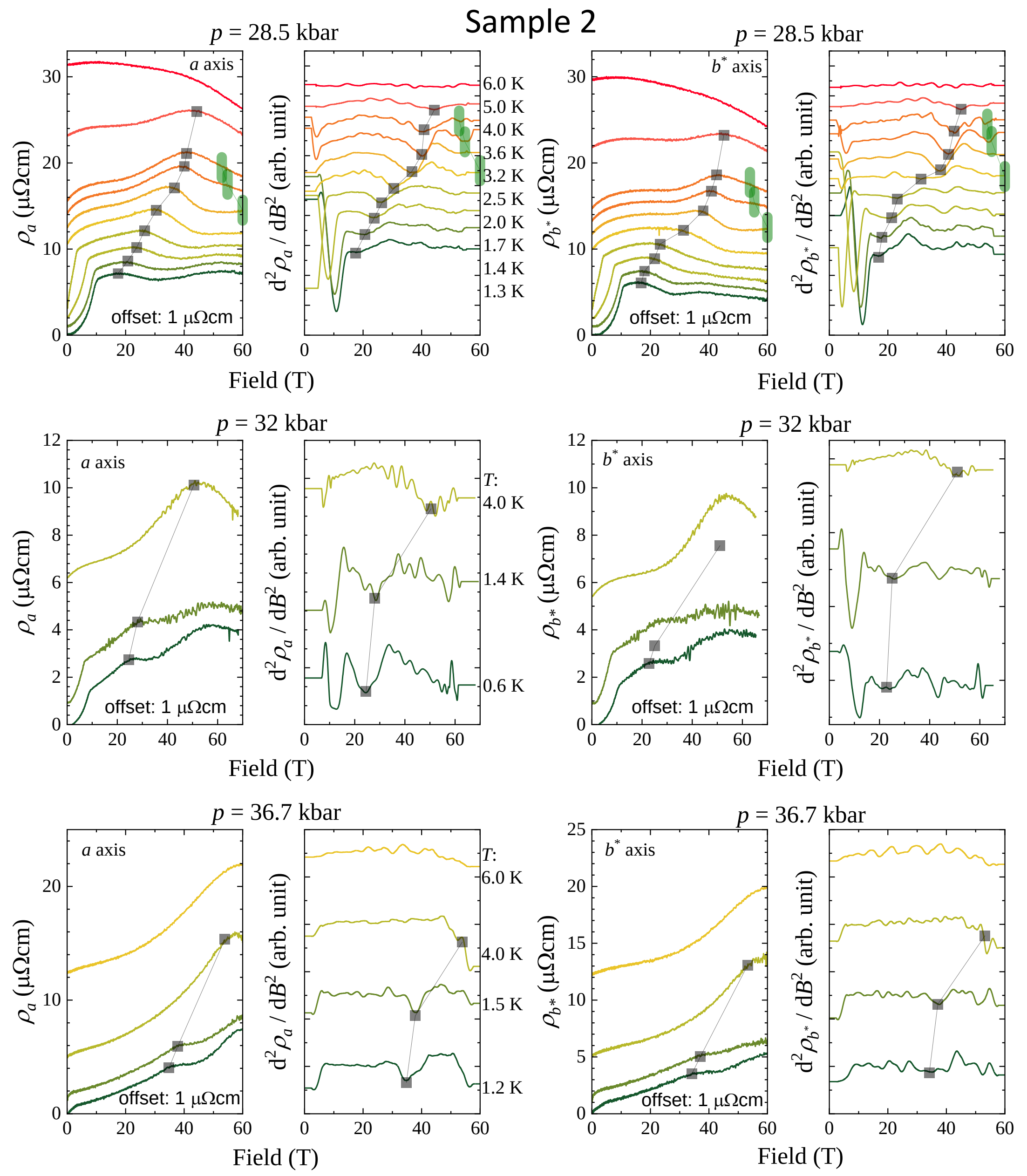

Supplementary Fig. 8. **Determination of** B_c **by second derivative analyses.** Magnetoresistivity for Sample 2 recorded for three pressures at different temperatures. Curves are shifted by a constant offset for clarity. **Left and right sides** are the field-down sweeps for $I||a$- and $I||b^*$-direction, respectively. **Right panels** show second derivatives of the data in the panel to their left. Green vertical dashes mark the AFM suppression field B_c. The steep increase in magnetoresistivity at low temperatures originates from pressure-induced superconductivity.

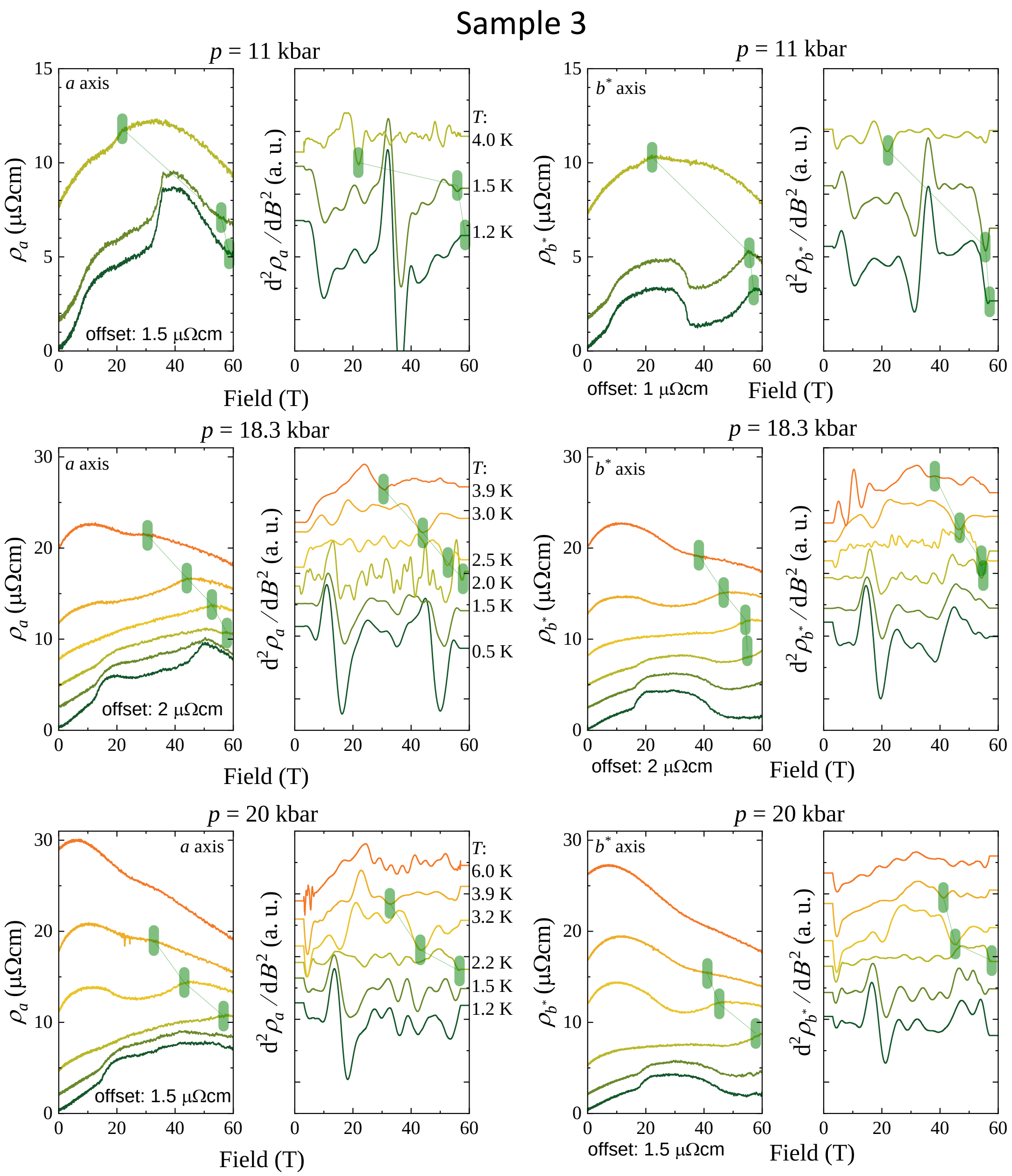

Supplementary Fig. 9. **Determination of** B_c **by second derivative analyses.** Magnetoresistivity for Sample 3 recorded for three pressures at different temperatures. Curves are shifted by a constant offset for clarity. **Left and right sides** are the field-down sweeps for $I||a$- and $I||b^*$-direction, respectively. **Right panels** show second derivatives of the data in the panel to their left. Green vertical dashes mark the AFM suppression field B_c.

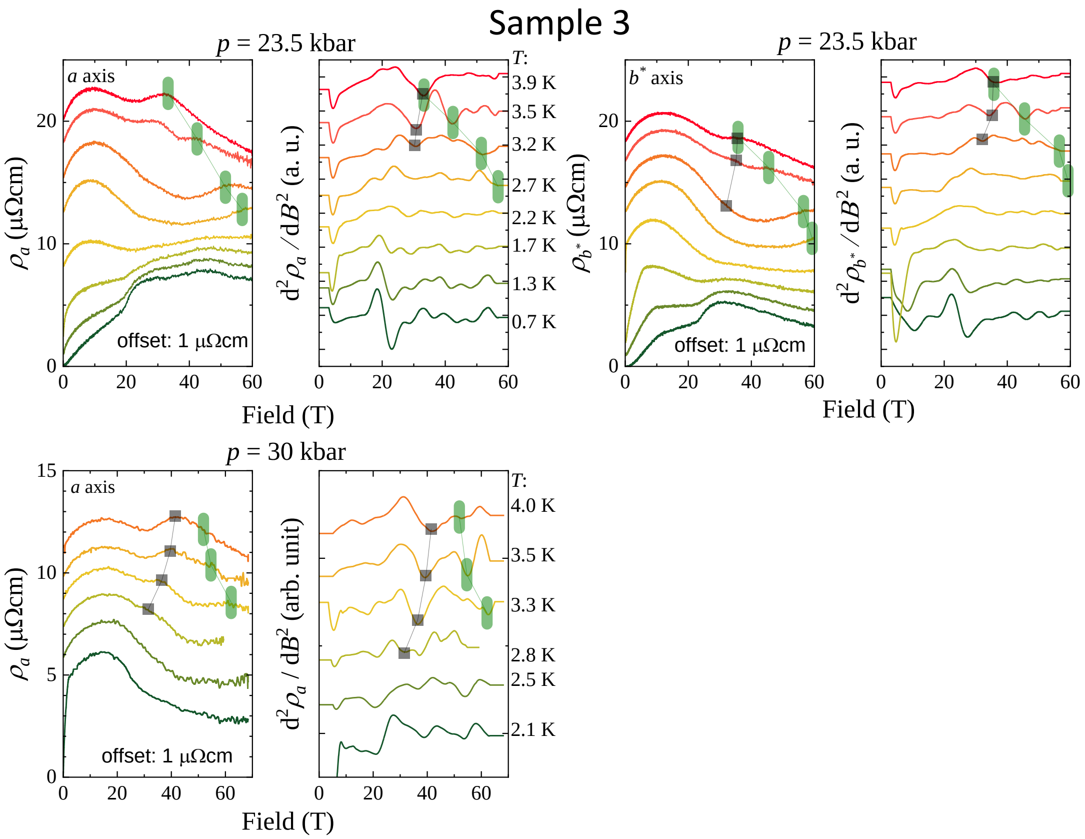

Supplementary Fig. 10. **Determination of B_c by second derivative analyses.** Magnetoresistivity for Sample 3 recorded for 2 pressures at different temperatures. Curves are shifted by a constant offset for clarity. **Left and right sides** are the field-down sweeps for $I||a$- and $I||b^*$-direction, respectively. **Right panels** show second derivatives of the data in the panel to their left. Green vertical dashes mark the AFM suppression field B_c. The steep increase in magnetoresistivity at low temperatures originates from pressure induced superconductivity.

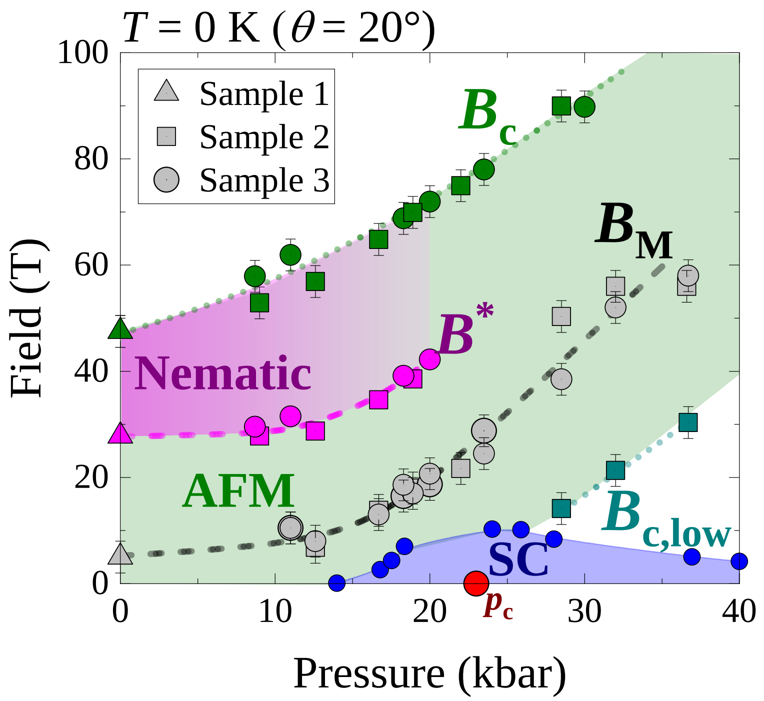

Supplementary Fig. 11. **Extrapolated zero-temperature** (p,B) **phase diagram of CeRhIn**$_5$ **for field tilted** 20° **off the** c **axis.** Here we summarize the phase diagram received by extrapolation of various values to zero temperature. The procedure of extrapolation is decribed in the main text. We combine here data from graphs shown in **Figures 3b, 6d, and f** in comparison to previously published results on the superconducting (SC) ground state by Knebel et al. [5] marked by blue dots. The diagram is comprised of base temperature values extrapolated to $T = 0\,\mathrm{K}$ for the magnetic order onset field $B_{\mathrm{c,low}}$ (dark cyan), the metamagnetic transition field B_{M} (grey), the nematic transition field B^* (magenta), and the antiferromagnetic suppression field B_{c}(green). p_{c} highlights the quantum critical point.

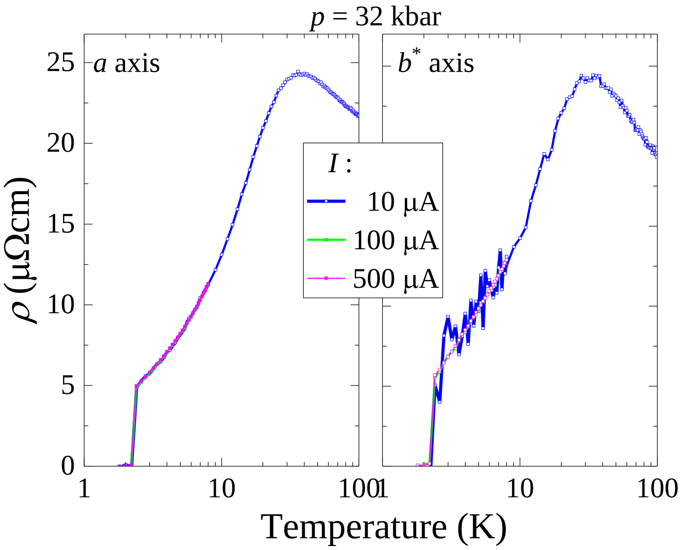

Supplementary Fig. 12. **Zero-field characterization.** Zero-field resistivity of $CeRhIn_5$ sample 2 at $p = 32$ kbar for both directions, $I||a$ and $I||b^*$, in the **left and right panel**, respectively. Data was recorded in a Quantum Design PPMS for three different currents. Superconductivity becomes visible at pressures larger than 20 kbar. Note: the current levels ($I \approx 400\,\mu$A) required to clearly resolve the resistive anomalies in the normal state result in an appreciable current density in the device ($j_c \approx 1 \times 10^3$ A/cm^2). For the chosen current, all the main high-field features, reported previously in zero-pressure experiments, were observed. It is evident that robust superconductivity is present in these devices above the critical pressure.

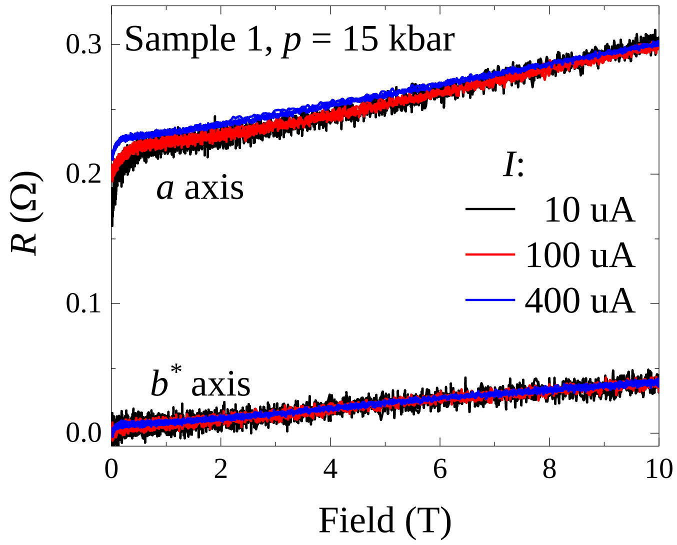

Supplementary Fig. 13. **Low-field characterization.** Example of low-field magnetoresistivity of $CeRhIn_5$, sample 1 at $T = 0.5$ T and $p = 15$ kbar recorded in the pulsed-magnet setup for both directions, $I||a$ and $I||b^*$. We verify ohmic behavior,i.e., no significant overheating related to the high current-density for the applied currents was observed during tests for low-field pulses under pressure.

SUPPLEMENTARY REFERENCES

* Corresponding author E-mail: t.helm@hzdr.de, philip.moll@epfl.ch

[1] Shishido, H., Settai, R., Harima, H. & Onuki, Y. A drastic change of the Fermi surface at a critical pressure in $CeRhIn_5$: dHvA study under pressure. *Journal of the Physical Society of Japan* **74**, 1103–1106 (2005). URL http://journals.jps.jp/doi/10.1143/JPSJ.74.1103.

[2] Ronning, F. *et al.* Electronic in-plane symmetry breaking at field-tuned quantum criticality in $CeRhIn_5$. *Nature* **548**, 313–317 (2017). URL http://www.nature.com/doifinder/10.1038/nature23315.

[3] Daubechies, I. *Ten Lectures on Wavelets* (Society for Industrial and Applied Mathematics, 1992). URL https://epubs.siam.org/doi/abs/10.1137/1.9781611970104. https://epubs.siam.org/doi/pdf/10.1137/1.9781611970104.

[4] Dautov, C. P. & Özerdem, M. S. Wavelet transform and signal denoising using wavelet method. In *2018 26th Signal Processing and Communications Applications Conference (SIU)*, 1–4 (2018).

[5] Knebel, G. *et al.* Antiferromagnetism and Superconductivity in $CeRhIn_5$. *Journal of the Physical Society of Japan* **80**, SA001 (2011). URL https://doi.org/10.1143/JPSJS.80SA.SA001.
